# Adipose Hypothermia in Obesity and Its Association with Period Homolog 1, Insulin Sensitivity, and Inflammation in Fat

**DOI:** 10.1371/journal.pone.0112813

**Published:** 2014-11-14

**Authors:** Masaya Yamaoka, Norikazu Maeda, Yasunori Takayama, Ryohei Sekimoto, Yu Tsushima, Keisuke Matsuda, Takuya Mori, Kana Inoue, Hitoshi Nishizawa, Makoto Tominaga, Tohru Funahashi, Iichiro Shimomura

**Affiliations:** 1 Department of Metabolic Medicine, Graduate School of Medicine, Osaka University, Suita, Osaka, 565-0871, Japan; 2 Division of Cell Signaling, Okazaki Institute for Integrative Bioscience (National Institute for Physiological Sciences), National Institutes of Natural Sciences, Okazaki, Aichi, 444-8787, Japan; 3 Department of Physiological Sciences, The Graduate University for Advanced Studies, Okazaki, Aichi, 444-8585, Japan; 4 Department of Metabolism and Atherosclerosis, Graduate School of Medicine, Osaka University, Suita, Osaka, 565-0871, Japan; Karlsruhe Institute of Technology, Germany

## Abstract

Visceral fat adiposity plays an important role in the development of metabolic syndrome. We reported previously the impact of human visceral fat adiposity on gene expression profile of peripheral blood cells. Genes related to circadian rhythm were highly associated with visceral fat area and period homolog 1 (PER1) showed the most significant negative correlation with visceral fat area. However, regulation of adipose Per1 remains poorly understood. The present study was designed to understand the regulation of Per1 in adipose tissues. Adipose Per1 mRNA levels of *ob/ob* mice were markedly low at 25 and 35 weeks of age. The levels of other core clock genes of white adipose tissues were also low in *ob/ob* mice at 25 and 35 weeks of age. Per1 mRNA was mainly expressed in the mature adipocyte fraction (MAF) and it was significantly low in MAF of *ob/ob* mice. To examine the possible mechanisms, 3T3-L1 adipocytes were treated with H_2_O_2_, tumor necrosis factor-α (TNF-α), S100A8, and lipopolysaccharide (LPS). However, no significant changes in Per1 mRNA level were observed by these agents. Exposure of cultured 3T3-L1 adipocytes to low temperature (33°C) decreased Per1 and catalase, and increased monocyte chemoattractant protein-1 (Mcp-1) mRNA levels. Hypothermia also worsened insulin-mediated Akt phosphorylation in 3T3-L1 adipocytes. Finally, telemetric analysis showed low temperature of adipose tissues in *ob/ob* mice. In obesity, adipose hypothermia seems to accelerate adipocyte dysfunction.

## Introduction

Visceral fat accumulation causes various metabolic disorders, collectively termed the metabolic syndrome, and is associated with the development of atherosclerosis [1], [2]. The molecular basis of the metabolic syndrome has been elucidated. Several pathological changes, such as dysregulation of adipocytokines [3], low-grade inflammation [4], excess reactive oxygen species (ROS) [5], and hypoxia [6], have been described in obese adipose tissue and these factors interactively initiate and accelerate the pathological condition of the metabolic syndrome.

We recently tested whether visceral fat adiposity impacts the gene expression profile of peripheral blood cells in human subjects [7], [8] and, in a series of exploratory research, identified several novel molecules involved in adipocyte biology [9], [10]. In the one clinical study, we demonstrated a significant correlation between genes related to the circadian rhythm and visceral fat area [7]. Among the genes related to the circadian rhythm, period homolog 1 (PER1) showed the most significant negative correlation with visceral fat area [7]. Increasing evidence points to a close association between the circadian clock oscillator and metabolic syndrome [11]–[13]. However, the precise link between circadian rhythm and metabolic syndrome remains obscure.

The heterodimer complex of brain and muscle ARNTL-like protein 1 (Bmal1) and circadian locomotor output cycles kaput (Clock) positively regulate the transcription of crytochrome (Cry) and period (Per). The heterodimers of Cry and Per suppress their own expression by binding to Bmal1/Clock at transcriptional levels [14]–[16]. Such transcriptional circuitries closely form circadian oscillations, and PER1 is one of the core clock genes. However, the regulation of PER1 has not been fully elucidated in adipose tissues and adipocytes. The present study was designed to determine the regulatory mechanisms of adipose Per1 in obesity and the possible relationship between adipose hypothermia and adipocyte function.

## Materials and Methods

### Animals

Male C57BL/6N (B6) mice and *ob/ob* mice were obtained from the Charles River Japan Inc. (Kanagawa, Japan) and maintained at 22°C under a 12:12-h light–dark cycle (lights on from 8:00 to 20:00) in the animal experimental facilities. For tissue distribution analysis, deeply-anesthetized 8-week-old male B6 mice were euthanized by bleeding from the inferior vena cava after 12 hrs of fasting, and various tissue samples were excised. Mice were euthanized under feeding condition and tissues such as liver, muscle, kidney and epididymal white adipose tissues (WAT) were excised at 8, 25 and 35 weeks of age. In general experiments, mice were anesthetized with an intraperitoneal injection of a mixture of medetomidine (0.3 mg/kg body weight), midazolam (4 mg/kg body weight) and butorphanol tartrate (5 mg/kg body weight). The experimental protocols were approved by the Ethics Review Committee for Animal Experimentation of Osaka University School of Medicine and Japan National Institute for Physiological Sciences. This study also conforms to the Guide for the Care and Use of Laboratory Animals published by the US National Institutes of Health.

### Telemetric recording of adipose temperature and spontaneous locomotor activity

Male B6 or *ob/ob* mice were implanted with a radiotransmitter (TA11TA-F10, Data Sciences International) intraperitoneally under anesthesia with 1.5% isoflurane and 98.5% oxygen using an inhalation anesthesia apparatus (DS Pharma Biomedical, Osaka, Japan) at 6 weeks of age. Body temperature was maintained using an electrical heating pad (Fine Science Tools, North Vancouver, Canada) during the implant operation. The radiotransmitter was tied to the abdominal muscle near epididymal fat with surgical silk suture (Ethicon). One-week after surgery, the transmitter signals was detected by a radio receiver and processed by the computer. Spontaneous locomotor activity and body temperature were measured at 10 weeks of age. Mice were maintained at 25°C under a 12:12-h light–dark cycle (lights on from 8:00 to 20:00). Rectal temperatures were recorded using a cannula type thermocouple probe, and a digital thermometer (Unique Medical, Tokyo, Japan) under isoflurane anesthesia on a radio receiver board where epididymal temperature was simultaneously recorded.

### Fractionation of WAT

WAT was minced in Krebs-Ringer buffer containing 120 mmol/L NaCl, 4 mmol/L KH_2_PO_4_, 1 mmol/L MgSO_4_, 1 mmol/L CaCl_2_, 10 mmol/L NaHCO_3_, 30 mmol/L HEPES, 20 mmol/L adenosine, and 4% (wt/vol) bovine serum albumin (Calbiochem, San Diego, CA). Tissue suspensions were centrifuged at 500×*g* for 5 min to remove erythrocytes and free leukocytes. Suspensions were incubated at 37°C for 20 min under continuous shaking after the addition of collagenase at a final concentration of 2 mg/mL. Cell suspension was filtered through a 250 µm filter and then spun at 300×*g* for 1 min to separate the floating mature adipocytes fraction (MAF) from the stromal vascular cell fraction (SVF). The fractionation and washing procedures were repeated twice with Krebs-Ringer buffer. Both fractions were finally washed with phosphate buffered saline (PBS) and subjected to quantitative real-time polymerase chain reaction (RT-PCR).

### 3T3-L1 cell cultures

3T3-L1 cells were maintained and differentiated as described previously [17]. Briefly, cells were grown to confluence and differentiated over 48 hrs by induction medium (Dulbecco's modified Eagle medium (DMEM) supplemented with 10% fetal calf serum (FCS) containing 0.5 mM of 1-methyl-3-isobutylxanthine, 1 µM of dexamethasone, and 5 µg/mL of insulin). After incubation with the induction medium for 48 hrs, the medium was changed to maintenance medium (DMEM supplemented with 10% FCS). 3T3-L1 adipocytes were treated with 10 ng/mL of tumor necrosis factor-α (TNF-α) or 50 µM of H_2_O_2_ for 24 hours on days 9 and 21 after differentiation, respectively. On day 7 after differentiation, 3T3-L1 adipocytes were treated with 1 and 10 µg/mL of S100A8 (Giotto Biotech; Firenze, Italy), or 0.1 and 1 µg/ml of lipopolysaccharide (LPS) (Sigma) for 24 hrs.

For the hypothermia experiment, the medium was changed on day 5 after 3T3-L1 adipocytes differentiation and the cells were exposed to low temperature (33°C). 3T3-L1 adipocytes were harvested at 48 hrs after hypothermia and were subjected to quantitative RT-PCR analysis and measurement of insulin signaling. For the evaluation of insulin signaling, after exposure to low temperature (33°C), the cells were treated with or without 1 nM of insulin for 10 min and subsequently harvested.

### Quantification of mRNA levels

Isolation of total RNA and production of cDNA were performed as described previously [18]. Real-time PCR was performed on the ViiATM 7 real-time PCR system (Life Technologies) using the THUNDERBIRD^™^ qPCR Mix (TOYOBO, Osaka, Japan) according to the instructions provided by the manufacturer. The results for each sample were normalized to the respective 36B4 mRNA levels. The primers used in this study are listed in Table 1.

**Table 1:** Primers used in real-time polymerase chain reaction.

Primer	forward	reverse
mouse Per1	5′-CCA GAT TGG TGG AGG TTA CTG AGT-3′	5′-GCG AGA GTC TTC TTG GAG CAG TAG -3′
mouse Per2	5′-CAG CCA CCC TGA AAA GGA-3′	5′-GTG AGG GAC ACC ACA CTC TC-3′
mouse Cry1	5′-CGG TGG AAA TTG CTC TCA-3′	5′-GGC ATC CTC TTC CTG ACT A-3′
mouse Cry2	5′- CCA AGT GCA TCA TTG GCG T -3′	5′- TGT TGA GCC GAC TAG TCT CTG C -3′
mouse Bmal1	5′- CGT CGG GAC AAA ATG AAC A -3′	5′- TTC TGT GTA TGG GTT GGT GG -3′
mouse Clock	5′-TTG TTA GGA TGA AGG TCA AAC AGG-3′	5′- CAC AGT CTC GTC TCT AAG GAA GGA A-3′
mouse Adiponectin	5′-GAT GGC AGA GAT GGC ACT CC-3′	5′-GAT GGC AGA GAT GGC ACT CC-3′
mouse PPARγ2	5′-AAC TCT GGG AGA TTC TCC TGT TGA-3′	5-TGG TAA TTT CTT GTG AAG TGC TCA TA-3′
mouse Mcp-1	5′-CCA CTC ACC TGC TGC TAC TCA T-3′	5′-TGG TGA TCC TCT TGT AGC TCT CC-3′
mouse Cox7a1	5′-CAG CGT CAT GGT CAG TCT GT-3′	5′- AGA AAA CCG TGT GGC AGA GA-3′
mouse Cox8b	5′-GAA CCA TGA AGC CAA CGA CT-3′	5′-GCG AAG TTC ACA GTG GTT CC-3′
mouse Catalase	5′-CCA GCG ACC AGA TGA AGC AG-3′	5′-CCA CTC TCT CAG GAA TCC GC-3′
mouse 36B4	5′-AAG CGC GTC CTG GCA TTG TCT-3′	5′-CCG CAG GGG CAG CAG TGG T-3′

### Western blotting

Preparation of protein extracts from tissues and cells was performed as described previously [19]. Ten µg of protein was subjected to 4–20% gradient SDS-PAGE gel and then transferred to a nitrocellulose membrane (GE Healthcare, Little Chalfont, UK). For immunoblotting, the membrane was incubated with 1∶1,000 dilution of anti-Phospho-Akt (Ser473) (#9271, Cell Signaling Technology, Danvers, MA) or anti-Akt (#9272, Cell Signaling Technology). The secondary antibodies were used according to the protocol recommended by the manufacturer. The signal was detected by using the enhanced chemiluminescence kit (GE Healthcare).

### Statistical analysis

All values were expressed as mean ± SEM. Differences between variables were tested for statistical significance using one-factor ANOVA and the unpaired Student's t-test. A *P* value less than 0.05 denoted the presence of a statistically significant difference.

## Results

### Changes in Per1 expression in obese adipose tissues

**Figure 1:**
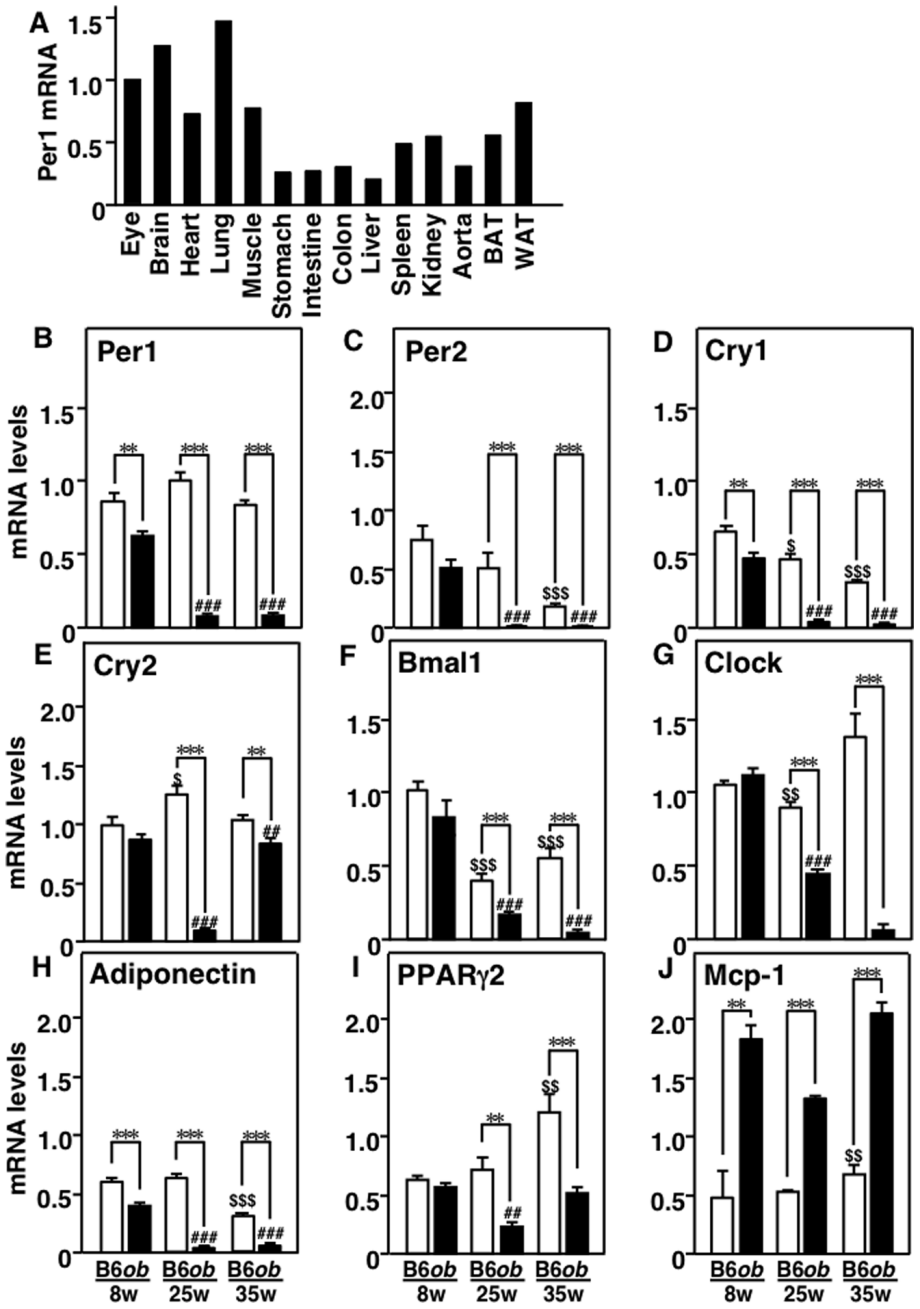
Core clock genes mRNA levels in obese adipose tissues. A, Tissue distribution of Per1 mRNA. The Per1 mRNA level of the eye was set at 1, and the mRNA levels of Per1 in other tissues are presented relative to the Per1 mRNA level of the eye. B to J, Changes in adipose core clock genes and adipose-related genes in C57BL/6N (B6) and *ob/ob* (*ob*) mice at 8, 25 and 35 weeks of age. n = 3–8 per group. Values are mean ± SEM. **P<0.01; ***P<0.001. ^$^P<0.05; ^$$^P<0.01; ^$$$^P<0.001, compared to 8-week-old B6 mice. ^##^P<0.01; ^###^P<0.001, compared to 8-week-old *ob* mice.

Tissue distribution of Per1 was investigated in 8-week-old B6 mice (Figure 1A). Per1 was ubiquitously expressed in various tissues and its mRNA was abundantly expressed in WAT.

WAT of B6 and *ob/ob* mice were harvested during the development of obesity and subjected to analysis of genes related to core clock circadian rhythm. Adipose Per1 mRNA levels were low at 8 weeks of age in *ob/ob* mice and markedly lower at 25 and 35 weeks of age, while the level of Per1 mRNA remained stable throughout the experiment in B6 mice (Figure 1B). Per2 and Cry1 mRNA levels in WAT gradually decreased in B6 mice with age, but the levels of these mRNAs decreased significantly in *ob/ob* mice at 25 and 35 weeks of age (Figure 1C and 1D). Similar changes were observed in other circadian rhythm genes, including Cry2, Bmal1, and Clock (Figure 1E–1G). Adiponectin mRNA level in WAT was significantly low in *ob/ob* mice at 8, 25, and 35 weeks of age (Figure 1H). Peroxisome proliferator-activated receptor-γ2 (PPARγ2) mRNA level in WAT gradually increased with age in B6 mice, whereas its mRNA level was significantly lower in *ob/ob* mice at 25 and 35 weeks of age (Figure 1I). Adipose monocyte chemoattractant protein-1 (Mcp-1) mRNA level was high in *ob/ob* mice at 8 weeks of age and further increased at 25 and 35 weeks of age (Figure 1J).

### Changes in core clock genes in other insulin-sensitive organs

**Figure 2:**
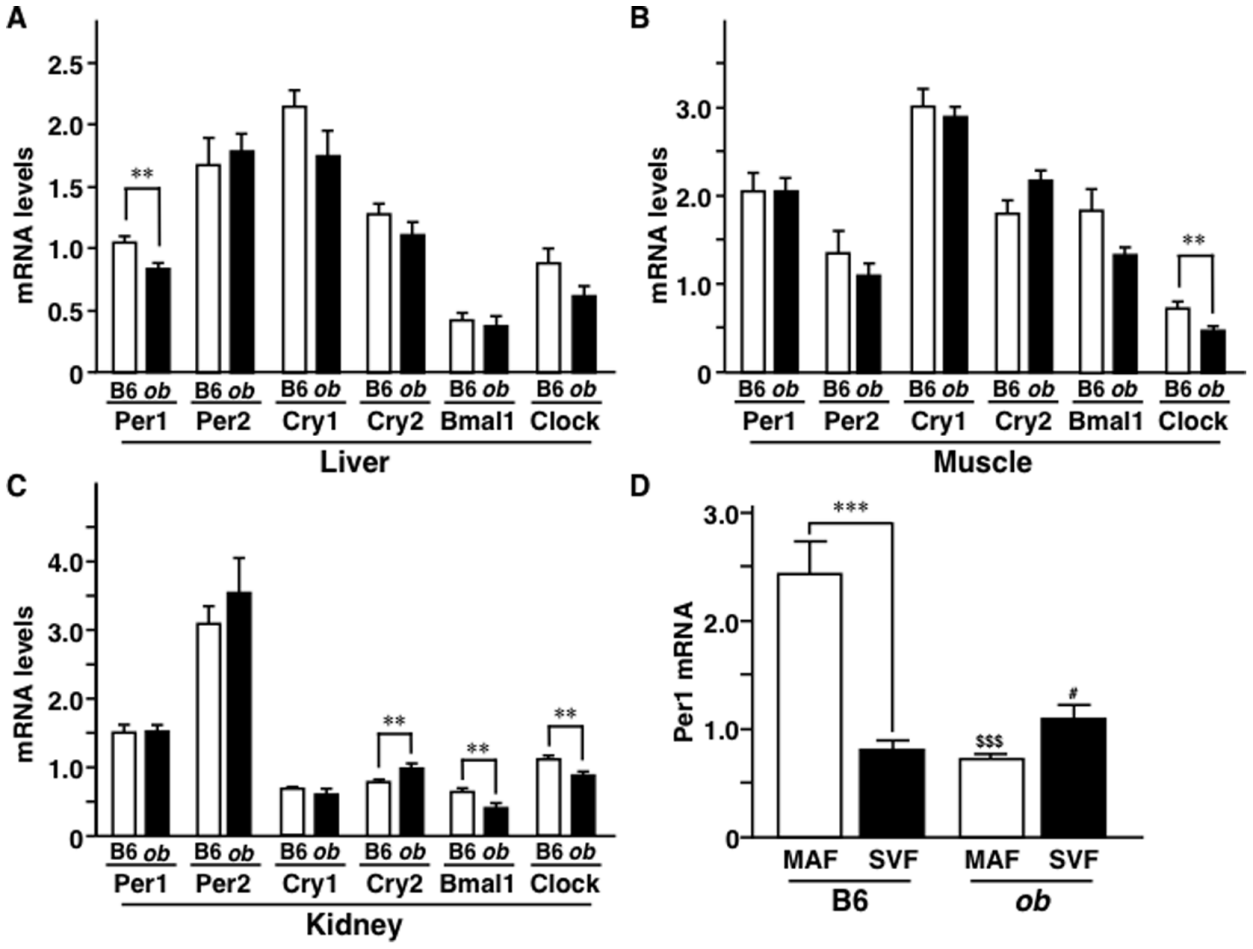
The mRNA expression levels of core clock genes. A to C, Comparison of core clock genes in the liver (A), muscle (B), and kidney (C) between C57BL/6N (B6) and *ob/ob* (*ob*) mice at 35 weeks of age. n = 3–8 per group. D, Per1 mRNA levels in mature adipocyte fraction (MAF) and stromal vascular fraction (SVF) of B6 and *ob* mice. WAT of 8-week-old mice was fractionated as described in Materials and Methods section. n = 6 per group. Values are mean ± SEM. **P<0.01; ***P<0.001. ^$$$^P<0.001, compared to MAF of B6 mice.^ #^P<0.05, compared to SVF of B6 mice.

As shown in Figure 1, the levels of the majority of core clock genes decreased dynamically in WAT at late stage of obesity. Next, we measured the mRNA levels of these clock genes in other insulin-sensitive organs, such as the liver, skeletal muscles, and kidneys at 35 weeks of age. In the liver, Per1 mRNA level was slightly lower in *ob/ob* mice compared to B6 mice while the levels of other circadian genes were comparable in the two species (Figure 2A). In skeletal muscles, Clock mRNA level was lower in *ob/ob* compared with B6 mice, but there were no significant differences in other circadian clock genes between the two species (Figure 2B). In the kidney, Cry2 mRNA level was higher while Bmal1 and Clock mRNA levels were lower in *ob/ob* mice compared to B6 mice (Figure 2C). Per1, Per2, and Cry1 mRNA levels in the kidneys were similar in the two mice groups (Figure 2C). While the mRNA expression levels of several core clock genes in the liver, skeletal muscles, and kidneys varied between the two mice groups, the variability was much smaller, compared to those in WAT (compare Figures 2A–2C and 1B–1G).

To examine the adipose cell types that expressed Per1, WAT was fractionated into mature adipocyte fraction (MAF) and stromal vascular fraction (SVF) (Figure 2D). Per1 mRNA level in B6 mice was mainly expressed in MAF compared to SVF. In *ob/ob* mice, adipose Per1 mRNA level was lower in MAF while it was higher in SVF, compared to B6 mice. These results suggest that mature adipocytes are mainly responsible for the low Per1 mRNA level in obese WAT.

### Regulation of Per1 and insulin sensitivity in 3T3-L1 adipocytes under hypothermia

**Figure 3:**
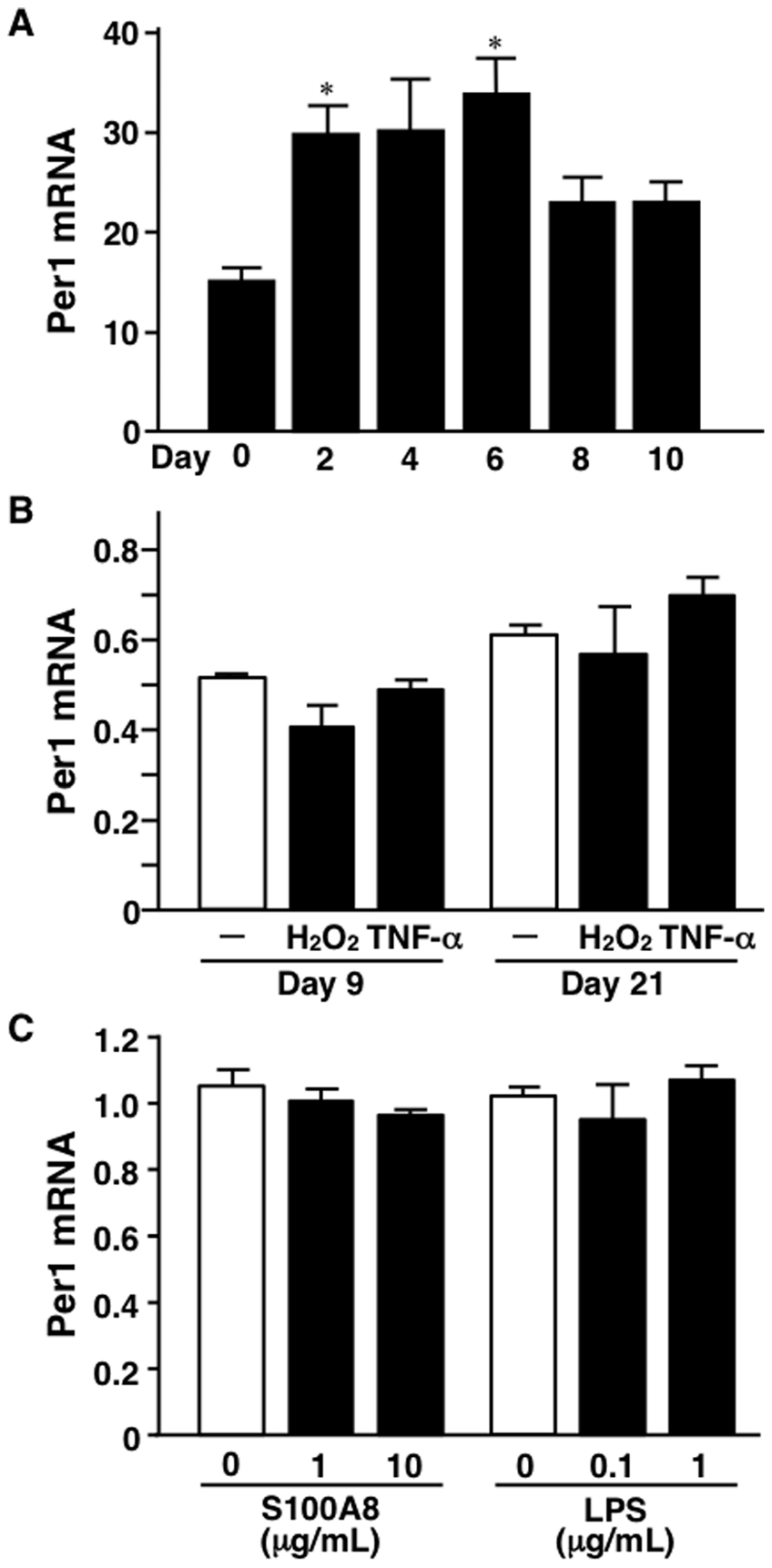
Effect of several factors on Per1 mRNA level in 3T3-L1 adipocytes. A, Changes in Per1 mRNA level during 3T3-L1 adipocyte differentiation. n = 3–6 per group. B, Effects of H_2_O_2_ and tumor necrosis factor-α (TNF-α) on Per1 mRNA level. 3T3-L1 adipocytes were treated with or without 10 ng/mL of TNF-α and 50 µM of H_2_O_2_ for 24 hours on days 9 and 21. n = 3 per group. C, Effect of S100A8 and LPS on Per1 mRNA level in 3T3-L1 adipocytes. 3T3-L1 adipocytes were treated with or without S100A8 and lipopolysaccharide (LPS) at the indicated concentrations for 24 hours. n = 4 per group. Values are mean ± SEM. *P<0.05, compared to Day 0.

Significantly low levels of Per1 mRNA were observed in MAF of *ob/ob* mice (Figure 2D). Next, we investigated the regulation of Per1 mRNA level in 3T3-L1 adipocytes. Induction of adipocyte differentiation increased Per1 mRNA level (Figure 3A). Incubation of 3T3-L1 adipocytes at day 9 and day 21 after 3T3-L1 adipocyte differentiation with either H_2_O_2_ or TNF-α (to mimic obese adipose tissues) (Figure 3B and 3C) did not result in any change in Per1 mRNA level (Figure 3B). Furthermore, neither S100A8 nor LPS, both of which activate TLR4-signaling and induce inflammation [20], altered Per1 mRNA expression level (Figure 3C).

**Figure 4:**
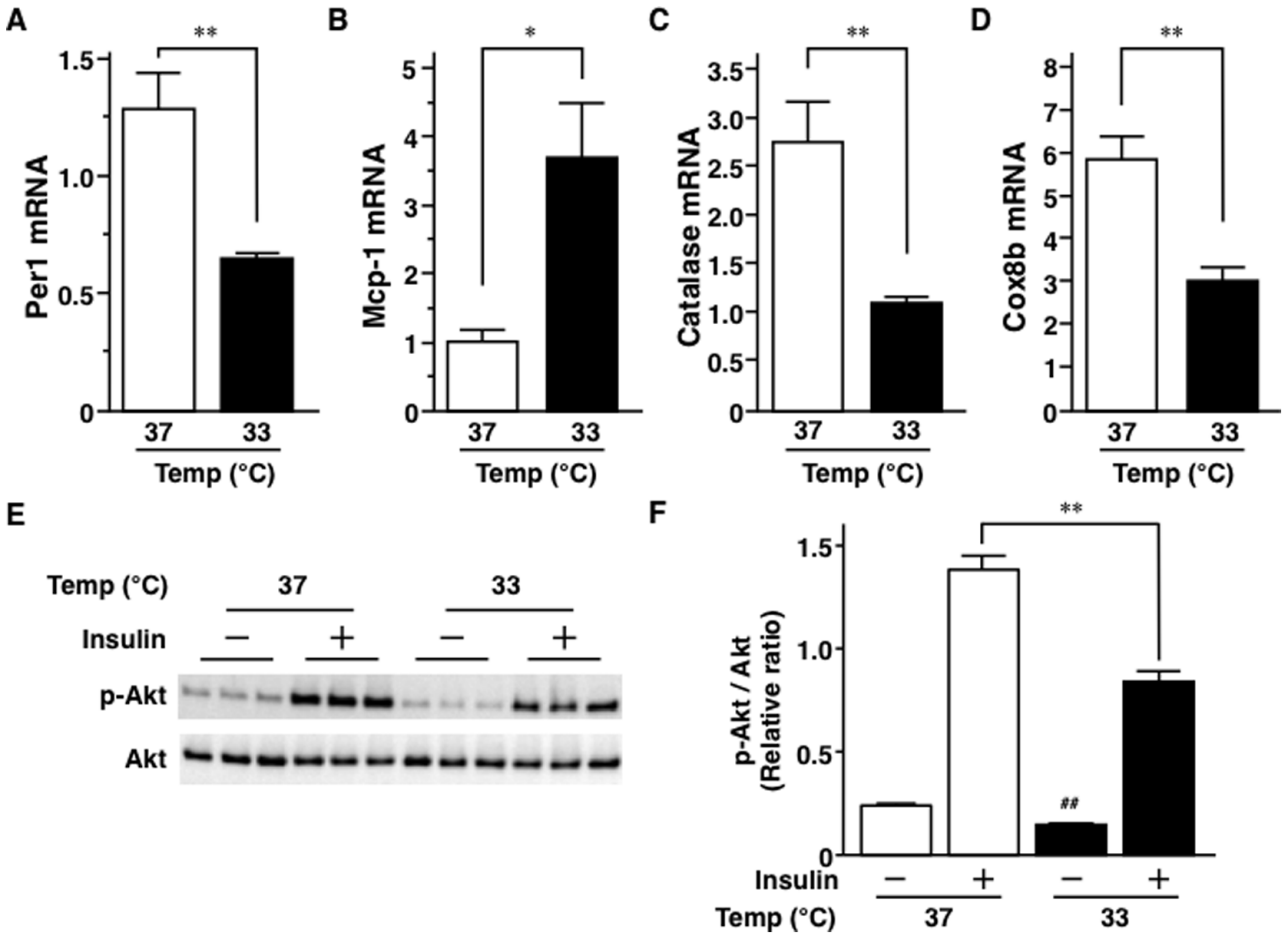
Effects of hypothermia on 3T3-L1 adipocytes. The culture medium was changed on day 5 after 3T3-L1 adipocyte differentiation and adipocytes were exposed to low temperature (33°C). 3T3-L1 adipocytes were collected at 0, 24, and 48 hrs after exposure to hypothermia. Hypothermia-induced changes in mRNA of Per1 (A), Mcp-1 (B), and Cox8b (C). D, Immunoblots of Akt and phospho-Akt. After exposure to low temperature (33°C) for 48 hrs, 3T3-L1 adipocytes were treated with or without 1 nM of insulin for 10 min and the cells were subjected to western blotting. E, Phosphorylation of Akt. In panels A to C, n = 6 per group and values are mean ± SEM. *P<0.05; **P<0.01. In panel E, n = 3 per group. Values are mean ± SEM. **P<0.01. ##P<0.01, compared to lane 1.

Body temperature is, in general, lower in obese mice [21]–[24], but the effect of hypothermia on adipocytes has not been elucidated. Exposure of 3T3-L1 adipocytes to hypothermia (culture at 33°C) resulted in a significant decrease in Per1 mRNA level (Figure 4A). On the other hand, hypothermia increased Mcp-1 mRNA level and lowered catalase mRNA level (Figure 4B and 4C). Furthermore, hypothermia significantly decreased the mRNA level of Cox8b, a thermo-sensitive gene [24] (Figure 4D).

We also examined the effect of hypothermia on insulin sensitivity of 3T3-L1 adipocytes. Interestingly, insulin-stimulated signals for phosphorylated-Akt (P-Akt) were faint in low temperature (33°C)-exposed 3T3-L1 adipocytes compared to those cultured at normal temperature (37°C) (Figure 4E). Phosphorylation of Akt was significantly poor in adipocytes exposed to hypothermia, irrespective of insulin stimulation (Figure 4F).

### Analysis of adipose temperature in mice

**Figure 5:**
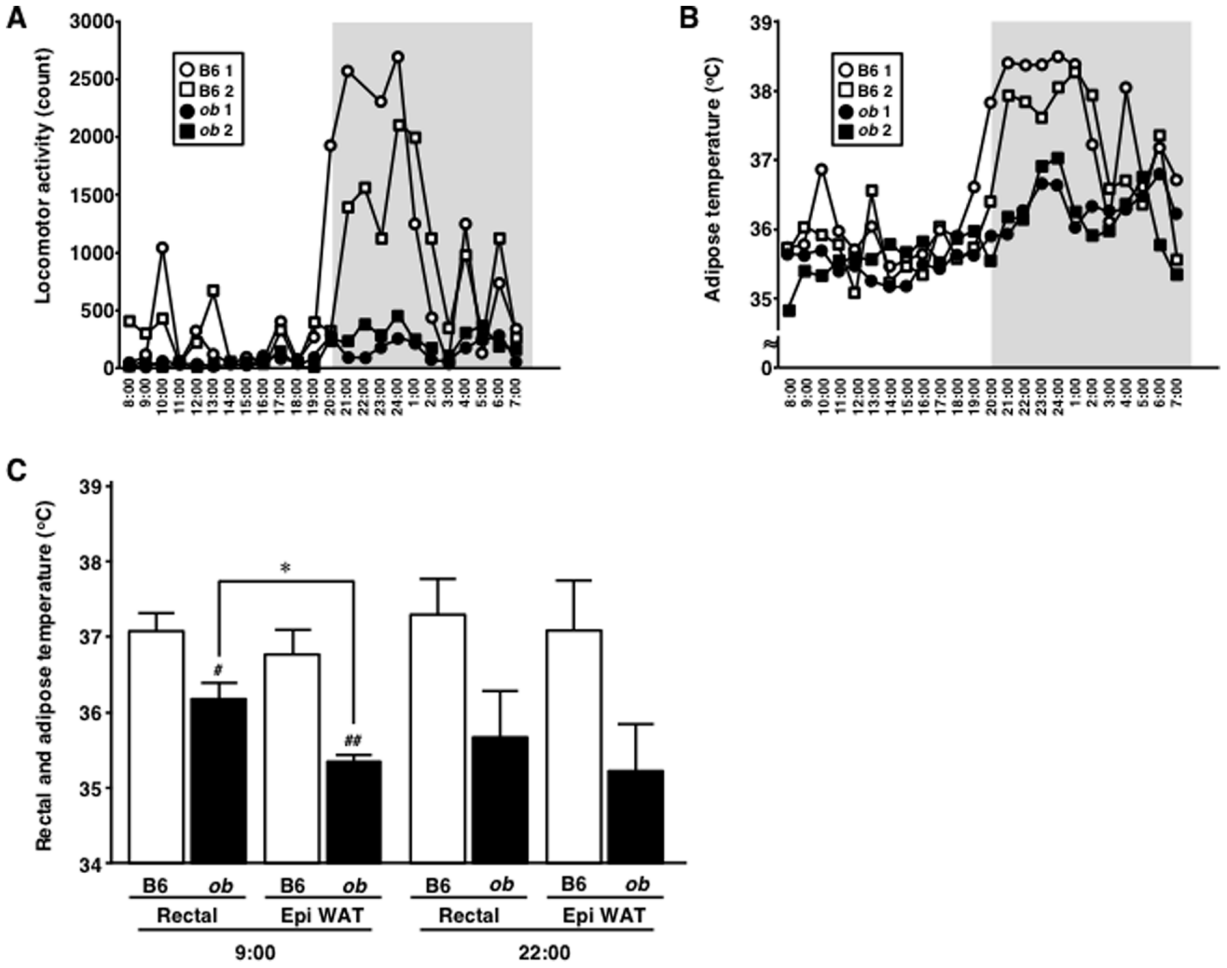
Analysis of adipose tissue temperature in mice. Telemetric recording of spontaneous locomotor activity (A) and epididymal fat temperature (B) was performed in C57BL/6N (B6) and *ob/ob* (*ob*) mice at 10 weeks of age. C, Comparison of rectal and adipose tissue temperature in B6 and *ob* mice. In panels A and B, n = 2 per group. In panel C, n = 4 per group. Values are mean ± SEM. *P<0.05. #P<0.05, ##P<0.01, compared to B6 in each group.

To our knowledge, there is no information on the temperature of WAT. In the last part of this study, telemetry was employed to record WAT temperature during spontaneous locomotor activity in 10-week-old mice. Locomotor activity was clearly reduced in *ob/ob* mice, compared to lean control mice (Figure 5A). WAT temperature was lower during the dark period in *ob/ob* mice, compared to the light-on period (Figure 5B). Figure 5C shows rectal and epididymal fat temperatures measured twice daily at lights on (9 am) and lights-off (10 pm) periods. During the light period, rectal and adipose temperatures were significantly lower in *ob/ob* mice than lean control mice. In general, the recorded temperatures in *ob/ob* mice tended to be lower than in B6 mice (Figure 5C, bar 5 versus 6, P = 0.082; bar 7 versus 8, P = 0.087). Interestingly, WAT temperature was significantly lower than rectal temperature in *ob/ob* mice during the light period (bar 2 versus 4). A similar trend was noted during the dark period, though the difference was not statistically significant.

## Discussion

The major findings of present study were: *1)* Low expression of circadian clock genes in the adipose tissues of obese mice. *2)* Significantly low Per1 mRNA level in obese mature adipocytes fraction. *3)* Low temperature significantly reduced Per1 mRNA level and reduced sensitivity to insulin in 3T3-L1 adipocytes. *4)* Adipose tissue temperature during physical activity was significantly lower in obese mice than the control.

We have recently reported the presence of a negative correlation between PER1 mRNA level in human peripheral blood and visceral fat area [7]. The present *in vivo* animal study showed a significant downregulation of adipose Per1 mRNA level in obesity. The results of a series of studies from our laboratory [11]–[13] suggested that the expression of Per1 gene in peripheral blood partly reflects visceral fat status. Furthermore, we also reported changes in the expression of various genes in adipose tissues of obese animals, and that genes related to inflammation, adipocytokines, and metabolism, are involved in the pathophysiology of obesity. Accumulating evidence indicates a close interrelationship between the circadian clock oscillator and metabolic syndrome [11]–[13]. Per-mutant mice developed obesity under high-fat diet [26] and blunted sleep disturbance-induced changes in the expression of genes related to the metabolic process [27], suggesting the impact of circadian clock genes on adipose metabolic response. However, since adipose-specific Per1 null mice are currently unavailable, the *in vivo* role of adipose Per1 remains poorly defined.

Several reports reported oscillations in Per1 and other core clock genes in WAT [28] and also under-expression of Per1 in WAT of *ob/ob* mice, compared to B6 mice [29]. Our results confirmed the latter finding. Other studies also reported that calorie restriction suppressed Per1 expression in WAT of B6 mice [30]. These results suggest that the nutritional status of the whole body could influence adipose Per1 expression. We examined the effect of insulin on Per1 mRNA level in 3T3-L1 adipocytes, but no consistent results were obtained (data not shown). Evidently, Bmal1 and Clock form a heterodimer complex and the Bmal1/Clock complex activates the transcription of Cry and Per. The heterodimers of Cry and Per negatively regulate their own expression by binding the Bmal1/Clock complex and inhibiting their transcriptional activities [14]–[16]. Such transcriptional circuit works precisely at the cellular level and generates circadian oscillation. Furthermore, several factors that affect core clock gene expressions have been reported. In liver and blood cells, Per1 mRNA level seems to be regulated by steroids [31]–[33], catecholamines [34]–[36], and hypoxia [37]. Bmal1 is positively regulated by PPARα in the liver [38] and by PPARγ in the vasculature [39]. However, there is little or no information on the regulation of Per1 in adipocytes and adipose tissues. As shown in Figure 3B and 3C, TNF-α, H_2_O_2_, or LPS failed to elicit significant changes in Per1 mRNA level in 3T3-L1 adipocytes, mimicking obese adipose tissues. Treatment with S100A8, a novel adipocytokine associated with visceral fat accumulation and atherosclerosis [8], [17], [18], did not alter Per1 mRNA level in 3T3-L1 adipocytes.

Body temperature of the obesity model mice has been reported to be lower than that of lean control mice [21]–[24]. To our knowledge, the effects of low temperature on 3T3-L1 adipocytes have not been examined. In the present study, we exposed cultured adipocytes to low temperature. Strikingly, exposure to low temperature reduced Per1 mRNA level in the presence of significant changes in thermo-sensitive genes [25], Cox8b (Figure 4C) and Cox7a1 (data not shown). The Spiegelman group has recently showed that the transient receptor potential vanilloid 4 (TRPV4) mediates both thermogenic and proinflammatory programs in adipocytes [25], suggesting a close molecular association between inflammation and temperature in these cells. Our results also showed that exposure of 3T3-L1 adipocytes to low temperature significantly increased Mcp-1 mRNA level and decreased catalase mRNA level (Figure 4B). One recent study demonstrated an increase in cell proliferation by the culture medium from hypothermia-incubated adipocytes [40]. These results suggest that adipose hypothermia may induce various growth factors, oxidative stresses, and cytokines, such as Mcp-1. Increasing evidence from analysis of genetically-engineered mice describes a close association between body temperature and obesity-related disorders, such as insulin resistance and inflammation [41]–[43]. Interestingly, one recent study reported that intermittent exposure of mice to cold increases fat accumulation and stimulates *de novo* lipogenesis [44], suggesting that hypothermia induces adipocyte hypertrophy and obesity. As shown in Figure 4E and 4F, exposure of adipocytes to hypothermia degraded their insulin sensitivity. Our group showed previously a state of hypoperfusion and hypoxia in adipose tissues of obese mice [6]. Several investigators have also demonstrated that hypothermia of local tissue is caused by local hypoperfusion [45]–[48]. As shown in Figure 5B, adipose temperature of *ob/ob* mice was low compared to B6 mice at night cycle, but it was not statistically different (Figure 5C), which could be explained by wide variation in the recorded temperature. The present study documented the presence of hypothermia in obese adipose tissues. Interestingly, adipose tissue temperature was significantly lower than rectal temperature in the same obese mice (Figure 5C), suggesting that adipose hypoperfusion accelerates low temperature in obese adipose tissue. Local hypoperfusion may accelerate adipose hypothermia and cause adipocyte dysfunction in obese adipose tissues. As shown in Figure 5A, low locomotor activity in obese mice may be one of the reasons for the low body temperature. At present, there is no direct evidence that local adipose tissue hypothermia causes adipose inflammation, insulin resistance, and dysregulation of clock gene expressions, because we cannot reduce the local temperature of WAT. Future technological advance in adipose biology should enhance our understanding of the pathological significance of adipose tissue hypothermia.

Taken together, present and previous studies suggest that adipose tissue hypothermia is one of the key factors linked to inflammation, insulin resistance, and disorders of circadian rhythm in obesity, although further investigation is needed in the future.
